# Supplementary material for: Pathobionts from chemically disrupted gut microbiota induce insulin-dependent diabetes in mice
Source: Microbiome. 2023 Mar 29;11:62. doi: 10.1186/s40168-023-01507-z (PMC10052834; doi:10.1186/s40168-023-01507-z)
Supplement: Supplementary file 2 — Additional file 1: Fig. S1. Fasting C-Peptide in the mice with and without 0.2% DSS. Fig. S2. Serum amylase (AMS) and serum lipase levels in each group. Fig. S3. 0.2% DSS increased food and water intake but did not change energy intake of mice. Fig. S4. 0.2% DSS did not change the lipid accumulation in mice. Fig. S5. 0.2% DSS did not impair gut barrier integrity and induce inflammation. Fig. S6. qPCR of 16S rRNA gene in fecal samples. Fig. S7. The gut microbiota had been depleted by more than 99% in the mice with a cocktail of antibiotics for 5 weeks. Fig. S8. The recipient mice developed a gut microbiota more similar to their donor mice. Fig. S9. Optimal classification performance of the sPLS-DA model of the gut microbial structure in NC and DSS mice. Fig. S10. Fluorescence in situ hybridization (FISH) of 16S rRNA in the pancreas of antibiotic treated mice. Fig. S11. Fluorescence in situ hybridization (FISH) of 16S rRNA in the pancreas in fecal microbiota transplanted mice. Fig. S12. The bacterial load in the liver as measured by real-time qPCR of 16S rRNA gene. Fig. S13. Flow cytometry evaluation of subtypes of leukocytes in the pancreas of FMNC and FMDSS mice. Fig. S14. 0.2% DSS did not enrich bacteria in pacreas and disrupt the immune tolerance in antibiotic treated mice. Fig. S15. Identification of Muribaculaceae strain MF 13079. Fig. S16. Functional annotation of flagella-related genes and motility test of MF13079. Fig. S17. Bacteria load in mesenteric lymph nodes (MLN) in Akk and Muri mice. Fig. S18. Bray-Curtis Distance based on 16S rRNA sequencing data of gut microbiota between recipient mice and human donors. Fig. S19. Fluorescence in situ hybridization (FISH) of 16S rRNA in the pancreas. Tables S1. Culture medium used for isolation of Muribaculeae. Table S2. COG in the genome of MF 13079. [file 40168_2023_1507_MOESM1_ESM.zip › TableS1.pdf]

Table S1. Culture medium used for isolation of Muribaculeae

Medium1 . MPYG medium with 3% FBS	
**Component**	**Concentration**
Trypticase peptone	5.00 g/L
Peptone	3.00 g/L
Peptone from soya	2.00 g/L
Polypeptone	1.00 g/L
Yeast extract	10.00 g/L
Beef extract	5.00 g/L
Glucose	5.00 g/L
Tween 80	0.50 ml/L
Maltose	0.50 g/L
Cellobiose	0.50 g/L
Starch, soluble	0.50 g/L
Glycerol	0.50 ml /L
K2HPO4	2.00 g/L
Cysteine-HCl x H2O	0.50 g/L
Na2S	0.25 g/L
Resazurin	1.00 mg/L
Salt solution (see below)	40.00 ml/L
Trace element(see below)	10.00 ml/L
Vitamin solution(see below)	10.00 ml/L
Haemin solution (see below)	10.00 ml/L
Vitamin K1 solution (see below)	0.20 ml/L
Salt solution(DSMZ Salt solution):	
CaCl2 x 2 H2O	0.25 g/L
MgSO4 x 7 H2O	0.50 g/L
K2HPO4	1.00 g/L
KH2PO4	1.00 g/L
NaHCO3	10.00 g/L
NaCl	2.00 g/L
Trace element solution(DSMZ Trace element solution):	
Nitrilotriacetic acid	1.50 g/L
MgSO4 x 7 H2O	3.00 g/L
MnSO4 x H2O	0.50 g/L
NaCl	1.00 g/L
FeSO4 x 7 H2O	0.10 g/L
CoSO4 x 7 H2O	0.18 g/L
CaCl2 x 2 H2O	0.10 g/L
ZnSO4 x 7 H2O	0.18 g/L
CuSO4 x 5 H2O	0.01 g/L
KAl(SO4)2 x 12 H2O	0.02 g/L
H3BO3	0.01 g/L
Na2MoO4 x 2 H2O	0.01 g/L
NiCl2 x 6 H2O	0.03 g/L
Na2SeO3 x 5 H2O	0.30 mg/L
Vitamin solution:	
Biotin	2.00 mg/L
Folic acid	2.00 mg/L
Pyridoxine-HCl	10.00 mg/L
Thiamine-HCl x 2 H2O	5.00 mg/L
Riboflavin	5.00 mg/L
Nicotinic acid	5.00 mg/L
D-Ca-pantothenate	5.00 mg/L
Vitamin B12	0.10 mg/L
p-Aminobenzoic acid	5.00 mg/L
Lipoic acid	5.00 mg/L
Haemin solution	
Dissolve 50 mg haemin in 1 ml 1 N NaOH; make up to 100 ml with distilled water. Store refrigerated.	
Vitamin K1 solution	
Dissolve 0.1 ml of vitamin K1 in 20 ml 95% ethanol and filter sterilize. Store refrigerated in a brown bottle.	
Others	
Fetal Bovine Serum	30 mL

Medium2 .BHI medium	
**Company**	**Product Code**
HOPEBIO Co., Ltd	HB8297

Medium3 .BHI medium with mucin	
**Company**	**Product Code**
HOPEBIO Co., Ltd	HB8297
**Conponents that need to be added**	
**Component**	**Concentration**
Mucin	4 g/L

Medium4 .BHI medium with DSS	
**Company**	**Product Code**
HOPEBIO Co., Ltd	HB8297
**Conponents that need to be added**	
**Component**	**Concentration**
DSS	1 g/L

Medium5 .BHI medium with acetic acid	
**Company**	**Product Code**
HOPEBIO Co., Ltd	HB8297
**Conponents that need to be added**	
**Component**	**Concentration**
acetic acid	33 mM

Medium6 .BHI medium with acetic acid, DSS and mucin	
**Company**	**Product Code**
HOPEBIO Co., Ltd	HB8297
**Conponents that need to be added**	
**Component**	**Concentration**
acetic acid	33 mM
DSS	1 g/L
Mucin	4 g/L

Medium7 .BHI medium with sulfate	
**Company**	**Product Code**
HOPEBIO Co., Ltd	HB8297
**Conponents that need to be added**	
**Component**	**Concentration**
Na2SO4	0.1 g/L
FeSO4	0.05 g/L
(NH4)2SO4	0.09 g/L

Medium8.M2GSC medium (30% CRF)	
**Component**	**Concentration**
Clarified rumen fluid (CRF)	300 g/L
Casitone	10 g/L
Yeast extract	2.5 g/L
Glucose	2 g/L
Cellobiose	2 g/L
Soluble starch	2 g/L
Cysteine	1 g/L
NaHCO3	4 g/L
KH2PO4	0.45 g/L
K2HPO4	0.45 g/L
(NH4)2SO4	0.9 g/L
NaCl	0.9 g/L
MgSO4·7H2O	0.09 g/L
CaCl2	0.09 g/L
Resazurin	1 g/L
Agar	13 g/L

Medium9.M2GSC medium (40% CRF)	
**Component**	**Concentration**
Clarified rumen fluid (CRF)	400 g/L
Casitone	10 g/L
Yeast extract	2.5 g/L
Glucose	2 g/L
Cellobiose	2 g/L
Soluble starch	2 g/L
Cysteine	1 g/L
NaHCO3	4 g/L
KH2PO4	0.45 g/L
K2HPO4	0.45 g/L
(NH4)2SO4	0.9 g/L
NaCl	0.9 g/L
MgSO4·7H2O	0.09 g/L
CaCl2	0.09 g/L
Resazurin	1 g/L
Agar	13 g/L

Medium10.M2GSC medium with mucin	
**Component**	**Concentration**
Clarified rumen fluid (CRF)	300 g/L
Casitone	10 g/L
Yeast extract	2.5 g/L
Glucose	2 g/L
Cellobiose	2 g/L
Soluble starch	2 g/L
Cysteine	1 g/L
NaHCO3	4 g/L
KH2PO4	0.45 g/L
K2HPO4	0.45 g/L
(NH4)2SO4	0.9 g/L
NaCl	0.9 g/L
MgSO4·7H2O	0.09 g/L
CaCl2	0.09 g/L
Resazurin	1 g/L
Agar	13 g/L
**Conponents that need to be added**	
**Component**	**Concentration**
Mucin	4 g/L

Medium11.M2GSC medium with DSS	
**Component**	**Concentration**
Clarified rumen fluid (CRF)	300 g/L
Casitone	10 g/L
Yeast extract	2.5 g/L
Glucose	2 g/L
Cellobiose	2 g/L
Soluble starch	2 g/L
Cysteine	1 g/L
NaHCO3	4 g/L
KH2PO4	0.45 g/L
K2HPO4	0.45 g/L
(NH4)2SO4	0.9 g/L
NaCl	0.9 g/L
MgSO4·7H2O	0.09 g/L
CaCl2	0.09 g/L
Resazurin	1 g/L
Agar	13 g/L
**Conponents that need to be added**	
**Component**	**Concentration**
DSS	1 g/L

Medium12.M2GSC medium with acedic acid	
**Component**	**Concentration**
Clarified rumen fluid (CRF)	400 g/L
Casitone	10 g/L
Yeast extract	2.5 g/L
Glucose	2 g/L
Cellobiose	2 g/L
Soluble starch	2 g/L
Cysteine	1 g/L
NaHCO3	4 g/L
KH2PO4	0.45 g/L
K2HPO4	0.45 g/L
(NH4)2SO4	0.9 g/L
NaCl	0.9 g/L
MgSO4·7H2O	0.09 g/L
CaCl2	0.09 g/L
Resazurin	1 g/L
Agar	13 g/L
**Conponents that need to be added**	
**Component**	**Concentration**
acetic acid	33 mM

Medium13.M2GSC medium with acetic acid, DSS and mucin	
**Component**	**Concentration**
Clarified rumen fluid (CRF)	300 g/L
Casitone	10 g/L
Yeast extract	2.5 g/L
Glucose	2 g/L
Cellobiose	2 g/L
Soluble starch	2 g/L
Cysteine	1 g/L
NaHCO3	4 g/L
KH2PO4	0.45 g/L
K2HPO4	0.45 g/L
(NH4)2SO4	0.9 g/L
NaCl	0.9 g/L
MgSO4·7H2O	0.09 g/L
CaCl2	0.09 g/L
Resazurin	1 g/L
Agar	13 g/L
**Conponents that need to be added**	
**Component**	**Concentration**
acetic acid	33 mM
DSS	1 g/L
Mucin	4 g/L

Medium14.M2GSC medium with sulfate	
**Component**	**Concentration**
Clarified rumen fluid (CRF)	400 g/L
Casitone	10 g/L
Yeast extract	2.5 g/L
Glucose	2 g/L
Cellobiose	2 g/L
Soluble starch	2 g/L
Cysteine	1 g/L
NaHCO3	4 g/L
KH2PO4	0.45 g/L
K2HPO4	0.45 g/L
(NH4)2SO4	0.9 g/L
NaCl	0.9 g/L
MgSO4·7H2O	0.09 g/L
CaCl2	0.09 g/L
Resazurin	1 g/L
Agar	13 g/L
**Conponents that need to be added**	
**Component**	**Concentration**
Na2SO4	0.1 g/L
FeSO4	0.05 g/L
(NH4)2SO4	0.09 g/L

Medium15.mGAM medium	
**Company**	**Product Code**
HOPEBIO Co., Ltd	HB8462

Medium16.mGAM medium with mucin	
**Company**	**Product Code**
HOPEBIO Co., Ltd	HB8462
**Conponents that need to be added**	
**Component**	**Concentration**
Mucin	4 g/L

Medium17.mGAM medium with DSS	
**Company**	**Product Code**
HOPEBIO Co., Ltd	HB8462
**Conponents that need to be added**	
**Component**	**Concentration**
DSS	1 g/L

Medium18.mGAM medium with acetic acid	
**Company**	**Product Code**
HOPEBIO Co., Ltd	HB8462
**Conponents that need to be added**	
**Component**	**Concentration**
acetic acid	33 mM

Medium19.mGAM medium with acetic acid, DSS and mucin	
**Company**	**Product Code**
HOPEBIO Co., Ltd	HB8462
**Conponents that need to be added**	
**Component**	**Concentration**
acetic acid	33 mM
DSS	1 g/L
Mucin	4 g/L

Medium20.mGAM medium with sulfate	
**Company**	**Product Code**
HOPEBIO Co., Ltd	HB8462
**Conponents that need to be added**	
**Component**	**Concentration**
Na2SO4	0.1 g/L
FeSO4	0.05 g/L
(NH4)2SO4	0.09 g/L

Medium21.mGAM medium with SCFAs	
**Company**	**Product Code**
HOPEBIO Co., Ltd	HB8462
**Conponents that need to be added**	
**Component**	**Concentration**
acetic acid	33 mM
propionic acid	8 mM
butyric acid	4 mM

Medium22.A2 medium	
**Component**	**Concentration**
BHI medium(HB8297, HOPEBIO Co., Ltd)	18.5 g/L
Yeast extract	5 g/L
TSB	15 g/L
K2HPO4	2.5 g/L
hemin	1 mg/L
glucose	1 g/L
L-cysteine	0.5 g/L
Na2CO3	0.4 g/L
VK3	1 mg/L
Fetal Bovine Serum	30 mL/L

Medium23. A2 medium with mucin	
**Component**	**Concentration**
BHI medium(HB8297, HOPEBIO Co., Ltd)	18.5 g/L
Yeast extract	5 g/L
TSB	15 g/L
K2HPO4	2.5 g/L
hemin	1 mg/L
glucose	1 g/L
L-cysteine	0.5 g/L
Na2CO3	0.4 g/L
VK3	1 mg/L
Fetal Bovine Serum	30 mL/L
**Conponents that need to be added**	
**Component**	**Concentration**
Mucin	4 g/L

Medium24. A2 medium with acetic acid	
**Component**	**Concentration**
BHI medium(HB8297, HOPEBIO Co., Ltd)	18.5 g/L
Yeast extract	5 g/L
TSB	15 g/L
K2HPO4	2.5 g/L
hemin	1 mg/L
glucose	1 g/L
L-cysteine	0.5 g/L
Na2CO3	0.4 g/L
VK3	1 mg/L
Fetal Bovine Serum	30 mL/L
**Conponents that need to be added**	
**Component**	**Concentration**
acetic acid	33 mM

Medium25.A2 medium with DSS	
**Component**	**Concentration**
BHI medium(HB8297, HOPEBIO Co., Ltd)	18.5 g/L
Yeast extract	5 g/L
TSB	15 g/L
K2HPO4	2.5 g/L
hemin	1 mg/L
glucose	1 g/L
L-cysteine	0.5 g/L
Na2CO3	0.4 g/L
VK3	1 mg/L
Fetal Bovine Serum	30 mL/L
**Conponents that need to be added**	
**Component**	**Concentration**
DSS	1 g/L

Medium26. A2 medium with acetic acid, DSS and mucin	
**Component**	**Concentration**
BHI medium(HB8297, HOPEBIO Co., Ltd)	18.5 g/L
Yeast extract	5 g/L
TSB	15 g/L
K2HPO4	2.5 g/L
hemin	1 mg/L
glucose	1 g/L
L-cysteine	0.5 g/L
Na2CO3	0.4 g/L
VK3	1 mg/L
Fetal Bovine Serum	30 mL/L
**Conponents that need to be added**	
**Component**	**Concentration**
acetic acid	33 mM
DSS	1 g/L
Mucin	4 g/L

Medium27. A2 medium with sulfate	
**Component**	**Concentration**
BHI medium(HB8297, HOPEBIO Co., Ltd)	18.5 g/L
Yeast extract	5 g/L
TSB	15 g/L
K2HPO4	2.5 g/L
hemin	1 mg/L
glucose	1 g/L
L-cysteine	0.5 g/L
Na2CO3	0.4 g/L
VK3	1 mg/L
Fetal Bovine Serum	30 mL/L
**Conponents that need to be added**	
**Component**	**Concentration**
Na2SO4	0.1 g/L
FeSO4	0.05 g/L
(NH4)2SO4	0.09 g/L

Medium28. A2 medium with SCFAs	
**Component**	**Concentration**
BHI medium(HB8297, HOPEBIO Co., Ltd)	18.5 g/L
Yeast extract	5 g/L
TSB	15 g/L
K2HPO4	2.5 g/L
hemin	1 mg/L
glucose	1 g/L
L-cysteine	0.5 g/L
Na2CO3	0.4 g/L
VK3	1 mg/L
Fetal Bovine Serum	30 mL/L
**Conponents that need to be added**	
**Component**	**Concentration**
acetic acid	33 mM
propionic acid	8 mM
butyric acid	4 mM

Medium29. BHI medium with SCFAs	
**Company**	**Product Code**
HOPEBIO Co., Ltd	HB8297
**Conponents that need to be added**	
**Component**	**Concentration**
acetic acid	33 mM
propionic acid	8 mM
butyric acid	4 mM

Medium30.Columbia Blood Agar medium	
**Company**	**Product Code**
HOPEBIO Co., Ltd	HB0124
**Conponents that need to be added**	
**Component**	**Concentration**
Off fiber sheep blood	50 mL/L

Medium31. Columbia Blood Agar medium with DSS	
**Company**	**Product Code**
HOPEBIO Co., Ltd	HB0124
**Conponents that need to be added**	
**Component**	**Concentration**
Off fiber sheep blood	50 mL/L
DSS	1 g/L

Medium32. Columbia Blood Agar medium with mucin	
**Company**	**Product Code**
HOPEBIO Co., Ltd	HB0124
**Conponents that need to be added**	
**Component**	**Concentration**
Off fiber sheep blood	50 mL/L
mucin	4 g/L

Medium33. Columbia Blood Agar medium with mucin and DSS	
**Company**	**Product Code**
HOPEBIO Co., Ltd	HB0124
**Conponents that need to be added**	
**Component**	**Concentration**
Off fiber sheep blood	50 mL/L
mucin	4 g/L
DSS	1 g/L

Medium34. MPYG medium	
**Component**	**Concentration**
Trypticase peptone	5.00 g/L
Peptone	3.00 g/L
Peptone from soya	2.00 g/L
Polypeptone	1.00 g/L
Yeast extract	10.00 g/L
Beef extract	5.00 g/L
Glucose	5.00 g/L
Tween 80	0.50 ml/L
Maltose	0.50 g/L
Cellobiose	0.50 g/L
Starch, soluble	0.50 g/L
Glycerol	0.50 ml /L
K2HPO4	2.00 g/L
Cysteine-HCl x H2O	0.50 g/L
Na2S	0.25 g/L
Resazurin	1.00 mg/L
Salt solution (see below)	40.00 ml/L
Trace element(see below)	10.00 ml/L
Vitamin solution(see below)	10.00 ml/L
Haemin solution (see below)	10.00 ml/L
Vitamin K1 solution (see below)	0.20 ml/L
Salt solution(DSMZ Salt solution):	
CaCl2 x 2 H2O	0.25 g/L
MgSO4 x 7 H2O	0.50 g/L
K2HPO4	1.00 g/L
KH2PO4	1.00 g/L
NaHCO3	10.00 g/L
NaCl	2.00 g/L
Trace element solution(DSMZ Trace element solution):	
Nitrilotriacetic acid	1.50 g/L
MgSO4 x 7 H2O	3.00 g/L
MnSO4 x H2O	0.50 g/L
NaCl	1.00 g/L
FeSO4 x 7 H2O	0.10 g/L
CoSO4 x 7 H2O	0.18 g/L
CaCl2 x 2 H2O	0.10 g/L
ZnSO4 x 7 H2O	0.18 g/L
CuSO4 x 5 H2O	0.01 g/L
KAl(SO4)2 x 12 H2O	0.02 g/L
H3BO3	0.01 g/L
Na2MoO4 x 2 H2O	0.01 g/L
NiCl2 x 6 H2O	0.03 g/L
Na2SeO3 x 5 H2O	0.30 mg/L
Vitamin solution:	
Biotin	2.00 mg/L
Folic acid	2.00 mg/L
Pyridoxine-HCl	10.00 mg/L
Thiamine-HCl x 2 H2O	5.00 mg/L
Riboflavin	5.00 mg/L
Nicotinic acid	5.00 mg/L
D-Ca-pantothenate	5.00 mg/L
Vitamin B12	0.10 mg/L
p-Aminobenzoic acid	5.00 mg/L
Lipoic acid	5.00 mg/L
Haemin solution	
Dissolve 50 mg haemin in 1 ml 1 N NaOH; make up to 100 ml with distilled water. Store refrigerated.	
Vitamin K1 solution	
Dissolve 0.1 ml of vitamin K1 in 20 ml 95% ethanol and filter sterilize. Store refrigerated in a brown bottle.	

Medium35. MPYG medium with mucin	
**Component**	**Concentration**
Trypticase peptone	5.00 g/L
Peptone	3.00 g/L
Peptone from soya	2.00 g/L
Polypeptone	1.00 g/L
Yeast extract	10.00 g/L
Beef extract	5.00 g/L
Glucose	5.00 g/L
Tween 80	0.50 ml/L
Maltose	0.50 g/L
Cellobiose	0.50 g/L
Starch, soluble	0.50 g/L
Glycerol	0.50 ml /L
K2HPO4	2.00 g/L
Cysteine-HCl x H2O	0.50 g/L
Na2S	0.25 g/L
Resazurin	1.00 mg/L
Salt solution (see below)	40.00 ml/L
Trace element(see below)	10.00 ml/L
Vitamin solution(see below)	10.00 ml/L
Haemin solution (see below)	10.00 ml/L
Vitamin K1 solution (see below)	0.20 ml/L
Salt solution(DSMZ Salt solution):	
CaCl2 x 2 H2O	0.25 g/L
MgSO4 x 7 H2O	0.50 g/L
K2HPO4	1.00 g/L
KH2PO4	1.00 g/L
NaHCO3	10.00 g/L
NaCl	2.00 g/L
Trace element solution(DSMZ Trace element solution):	
Nitrilotriacetic acid	1.50 g/L
MgSO4 x 7 H2O	3.00 g/L
MnSO4 x H2O	0.50 g/L
NaCl	1.00 g/L
FeSO4 x 7 H2O	0.10 g/L
CoSO4 x 7 H2O	0.18 g/L
CaCl2 x 2 H2O	0.10 g/L
ZnSO4 x 7 H2O	0.18 g/L
CuSO4 x 5 H2O	0.01 g/L
KAl(SO4)2 x 12 H2O	0.02 g/L
H3BO3	0.01 g/L
Na2MoO4 x 2 H2O	0.01 g/L
NiCl2 x 6 H2O	0.03 g/L
Na2SeO3 x 5 H2O	0.30 mg/L
Vitamin solution:	
Biotin	2.00 mg/L
Folic acid	2.00 mg/L
Pyridoxine-HCl	10.00 mg/L
Thiamine-HCl x 2 H2O	5.00 mg/L
Riboflavin	5.00 mg/L
Nicotinic acid	5.00 mg/L
D-Ca-pantothenate	5.00 mg/L
Vitamin B12	0.10 mg/L
p-Aminobenzoic acid	5.00 mg/L
Lipoic acid	5.00 mg/L
Haemin solution	
Dissolve 50 mg haemin in 1 ml 1 N NaOH; make up to 100 ml with distilled water. Store refrigerated.	
Vitamin K1 solution	
Dissolve 0.1 ml of vitamin K1 in 20 ml 95% ethanol and filter sterilize. Store refrigerated in a brown bottle.	
Others	
mucin	4 g/L

Medium36. MPYG medium with DSS	
**ComponentAAB3:AC61**	**Concentration**
Trypticase peptone	5.00 g/L
Peptone	3.00 g/L
Peptone from soya	2.00 g/L
Polypeptone	1.00 g/L
Yeast extract	10.00 g/L
Beef extract	5.00 g/L
Glucose	5.00 g/L
Tween 80	0.50 ml/L
Maltose	0.50 g/L
Cellobiose	0.50 g/L
Starch, soluble	0.50 g/L
Glycerol	0.50 ml /L
K2HPO4	2.00 g/L
Cysteine-HCl x H2O	0.50 g/L
Na2S	0.25 g/L
Resazurin	1.00 mg/L
Salt solution (see below)	40.00 ml/L
Trace element(see below)	10.00 ml/L
Vitamin solution(see below)	10.00 ml/L
Haemin solution (see below)	10.00 ml/L
Vitamin K1 solution (see below)	0.20 ml/L
Salt solution(DSMZ Salt solution):	
CaCl2 x 2 H2O	0.25 g/L
MgSO4 x 7 H2O	0.50 g/L
K2HPO4	1.00 g/L
KH2PO4	1.00 g/L
NaHCO3	10.00 g/L
NaCl	2.00 g/L
Trace element solution(DSMZ Trace element solution):	
Nitrilotriacetic acid	1.50 g/L
MgSO4 x 7 H2O	3.00 g/L
MnSO4 x H2O	0.50 g/L
NaCl	1.00 g/L
FeSO4 x 7 H2O	0.10 g/L
CoSO4 x 7 H2O	0.18 g/L
CaCl2 x 2 H2O	0.10 g/L
ZnSO4 x 7 H2O	0.18 g/L
CuSO4 x 5 H2O	0.01 g/L
KAl(SO4)2 x 12 H2O	0.02 g/L
H3BO3	0.01 g/L
Na2MoO4 x 2 H2O	0.01 g/L
NiCl2 x 6 H2O	0.03 g/L
Na2SeO3 x 5 H2O	0.30 mg/L
Vitamin solution:	
Biotin	2.00 mg/L
Folic acid	2.00 mg/L
Pyridoxine-HCl	10.00 mg/L
Thiamine-HCl x 2 H2O	5.00 mg/L
Riboflavin	5.00 mg/L
Nicotinic acid	5.00 mg/L
D-Ca-pantothenate	5.00 mg/L
Vitamin B12	0.10 mg/L
p-Aminobenzoic acid	5.00 mg/L
Lipoic acid	5.00 mg/L
Haemin solution	
Dissolve 50 mg haemin in 1 ml 1 N NaOH; make up to 100 ml with distilled water. Store refrigerated.	
Vitamin K1 solution	
Dissolve 0.1 ml of vitamin K1 in 20 ml 95% ethanol and filter sterilize. Store refrigerated in a brown bottle.	
Others	
DSS	1 g/L

Medium37. MPYG medium with acetic acid	
**Component**	**Concentration**
Trypticase peptone	5.00 g/L
Peptone	3.00 g/L
Peptone from soya	2.00 g/L
Polypeptone	1.00 g/L
Yeast extract	10.00 g/L
Beef extract	5.00 g/L
Glucose	5.00 g/L
Tween 80	0.50 ml/L
Maltose	0.50 g/L
Cellobiose	0.50 g/L
Starch, soluble	0.50 g/L
Glycerol	0.50 ml /L
K2HPO4	2.00 g/L
Cysteine-HCl x H2O	0.50 g/L
Na2S	0.25 g/L
Resazurin	1.00 mg/L
Salt solution (see below)	40.00 ml/L
Trace element(see below)	10.00 ml/L
Vitamin solution(see below)	10.00 ml/L
Haemin solution (see below)	10.00 ml/L
Vitamin K1 solution (see below)	0.20 ml/L
Salt solution(DSMZ Salt solution):	
CaCl2 x 2 H2O	0.25 g/L
MgSO4 x 7 H2O	0.50 g/L
K2HPO4	1.00 g/L
KH2PO4	1.00 g/L
NaHCO3	10.00 g/L
NaCl	2.00 g/L
Trace element solution(DSMZ Trace element solution):	
Nitrilotriacetic acid	1.50 g/L
MgSO4 x 7 H2O	3.00 g/L
MnSO4 x H2O	0.50 g/L
NaCl	1.00 g/L
FeSO4 x 7 H2O	0.10 g/L
CoSO4 x 7 H2O	0.18 g/L
CaCl2 x 2 H2O	0.10 g/L
ZnSO4 x 7 H2O	0.18 g/L
CuSO4 x 5 H2O	0.01 g/L
KAl(SO4)2 x 12 H2O	0.02 g/L
H3BO3	0.01 g/L
Na2MoO4 x 2 H2O	0.01 g/L
NiCl2 x 6 H2O	0.03 g/L
Na2SeO3 x 5 H2O	0.30 mg/L
Vitamin solution:	
Biotin	2.00 mg/L
Folic acid	2.00 mg/L
Pyridoxine-HCl	10.00 mg/L
Thiamine-HCl x 2 H2O	5.00 mg/L
Riboflavin	5.00 mg/L
Nicotinic acid	5.00 mg/L
D-Ca-pantothenate	5.00 mg/L
Vitamin B12	0.10 mg/L
p-Aminobenzoic acid	5.00 mg/L
Lipoic acid	5.00 mg/L
Haemin solution	
Dissolve 50 mg haemin in 1 ml 1 N NaOH; make up to 100 ml with distilled water. Store refrigerated.	
Vitamin K1 solution	
Dissolve 0.1 ml of vitamin K1 in 20 ml 95% ethanol and filter sterilize. Store refrigerated in a brown bottle.	
Others	
acetic acid	33 mM

Medium38. MPYG medium with mucin, DSS and acetic acid	
**Component**	**Concentration**
Trypticase peptone	5.00 g/L
Peptone	3.00 g/L
Peptone from soya	2.00 g/L
Polypeptone	1.00 g/L
Yeast extract	10.00 g/L
Beef extract	5.00 g/L
Glucose	5.00 g/L
Tween 80	0.50 ml/L
Maltose	0.50 g/L
Cellobiose	0.50 g/L
Starch, soluble	0.50 g/L
Glycerol	0.50 ml /L
K2HPO4	2.00 g/L
Cysteine-HCl x H2O	0.50 g/L
Na2S	0.25 g/L
Resazurin	1.00 mg/L
Salt solution (see below)	40.00 ml/L
Trace element(see below)	10.00 ml/L
Vitamin solution(see below)	10.00 ml/L
Haemin solution (see below)	10.00 ml/L
Vitamin K1 solution (see below)	0.20 ml/L
Salt solution(DSMZ Salt solution):	
CaCl2 x 2 H2O	0.25 g/L
MgSO4 x 7 H2O	0.50 g/L
K2HPO4	1.00 g/L
KH2PO4	1.00 g/L
NaHCO3	10.00 g/L
NaCl	2.00 g/L
Trace element solution(DSMZ Trace element solution):	
Nitrilotriacetic acid	1.50 g/L
MgSO4 x 7 H2O	3.00 g/L
MnSO4 x H2O	0.50 g/L
NaCl	1.00 g/L
FeSO4 x 7 H2O	0.10 g/L
CoSO4 x 7 H2O	0.18 g/L
CaCl2 x 2 H2O	0.10 g/L
ZnSO4 x 7 H2O	0.18 g/L
CuSO4 x 5 H2O	0.01 g/L
KAl(SO4)2 x 12 H2O	0.02 g/L
H3BO3	0.01 g/L
Na2MoO4 x 2 H2O	0.01 g/L
NiCl2 x 6 H2O	0.03 g/L
Na2SeO3 x 5 H2O	0.30 mg/L
Vitamin solution:	
Biotin	2.00 mg/L
Folic acid	2.00 mg/L
Pyridoxine-HCl	10.00 mg/L
Thiamine-HCl x 2 H2O	5.00 mg/L
Riboflavin	5.00 mg/L
Nicotinic acid	5.00 mg/L
D-Ca-pantothenate	5.00 mg/L
Vitamin B12	0.10 mg/L
p-Aminobenzoic acid	5.00 mg/L
Lipoic acid	5.00 mg/L
Haemin solution	
Dissolve 50 mg haemin in 1 ml 1 N NaOH; make up to 100 ml with distilled water. Store refrigerated.	
Vitamin K1 solution	
Dissolve 0.1 ml of vitamin K1 in 20 ml 95% ethanol and filter sterilize. Store refrigerated in a brown bottle.	
Others	
mucin	4 g/L
DSS	1 g/L
acetic acid	33 mM

Medium39. MPYG medium with sulfate	
**Component**	**Concentration**
Trypticase peptone	5.00 g/L
Peptone	3.00 g/L
Peptone from soya	2.00 g/L
Polypeptone	1.00 g/L
Yeast extract	10.00 g/L
Beef extract	5.00 g/L
Glucose	5.00 g/L
Tween 80	0.50 ml/L
Maltose	0.50 g/L
Cellobiose	0.50 g/L
Starch, soluble	0.50 g/L
Glycerol	0.50 ml /L
K2HPO4	2.00 g/L
Cysteine-HCl x H2O	0.50 g/L
Na2S	0.25 g/L
Resazurin	1.00 mg/L
Salt solution (see below)	40.00 ml/L
Trace element(see below)	10.00 ml/L
Vitamin solution(see below)	10.00 ml/L
Haemin solution (see below)	10.00 ml/L
Vitamin K1 solution (see below)	0.20 ml/L
Salt solution(DSMZ Salt solution):	
CaCl2 x 2 H2O	0.25 g/L
MgSO4 x 7 H2O	0.50 g/L
K2HPO4	1.00 g/L
KH2PO4	1.00 g/L
NaHCO3	10.00 g/L
NaCl	2.00 g/L
Trace element solution(DSMZ Trace element solution):	
Nitrilotriacetic acid	1.50 g/L
MgSO4 x 7 H2O	3.00 g/L
MnSO4 x H2O	0.50 g/L
NaCl	1.00 g/L
FeSO4 x 7 H2O	0.10 g/L
CoSO4 x 7 H2O	0.18 g/L
CaCl2 x 2 H2O	0.10 g/L
ZnSO4 x 7 H2O	0.18 g/L
CuSO4 x 5 H2O	0.01 g/L
KAl(SO4)2 x 12 H2O	0.02 g/L
H3BO3	0.01 g/L
Na2MoO4 x 2 H2O	0.01 g/L
NiCl2 x 6 H2O	0.03 g/L
Na2SeO3 x 5 H2O	0.30 mg/L
Vitamin solution:	
Biotin	2.00 mg/L
Folic acid	2.00 mg/L
Pyridoxine-HCl	10.00 mg/L
Thiamine-HCl x 2 H2O	5.00 mg/L
Riboflavin	5.00 mg/L
Nicotinic acid	5.00 mg/L
D-Ca-pantothenate	5.00 mg/L
Vitamin B12	0.10 mg/L
p-Aminobenzoic acid	5.00 mg/L
Lipoic acid	5.00 mg/L
Haemin solution	
Dissolve 50 mg haemin in 1 ml 1 N NaOH; make up to 100 ml with distilled water. Store refrigerated.	
Vitamin K1 solution	
Dissolve 0.1 ml of vitamin K1 in 20 ml 95% ethanol and filter sterilize. Store refrigerated in a brown bottle.	
Others	
Na2SO4	0.1 g/L
FeSO4	0.05 g/L
(NH4)2SO4	0.09 g/L

Medium40. MPYG medium with SCFAs	
**Component**	**Concentration**
Trypticase peptone	5.00 g/L
Peptone	3.00 g/L
Peptone from soya	2.00 g/L
Polypeptone	1.00 g/L
Yeast extract	10.00 g/L
Beef extract	5.00 g/L
Glucose	5.00 g/L
Tween 80	0.50 ml/L
Maltose	0.50 g/L
Cellobiose	0.50 g/L
Starch, soluble	0.50 g/L
Glycerol	0.50 ml /L
K2HPO4	2.00 g/L
Cysteine-HCl x H2O	0.50 g/L
Na2S	0.25 g/L
Resazurin	1.00 mg/L
Salt solution (see below)	40.00 ml/L
Trace element(see below)	10.00 ml/L
Vitamin solution(see below)	10.00 ml/L
Haemin solution (see below)	10.00 ml/L
Vitamin K1 solution (see below)	0.20 ml/L
Salt solution(DSMZ Salt solution):	
CaCl2 x 2 H2O	0.25 g/L
MgSO4 x 7 H2O	0.50 g/L
K2HPO4	1.00 g/L
KH2PO4	1.00 g/L
NaHCO3	10.00 g/L
NaCl	2.00 g/L
Trace element solution(DSMZ Trace element solution):	
Nitrilotriacetic acid	1.50 g/L
MgSO4 x 7 H2O	3.00 g/L
MnSO4 x H2O	0.50 g/L
NaCl	1.00 g/L
FeSO4 x 7 H2O	0.10 g/L
CoSO4 x 7 H2O	0.18 g/L
CaCl2 x 2 H2O	0.10 g/L
ZnSO4 x 7 H2O	0.18 g/L
CuSO4 x 5 H2O	0.01 g/L
KAl(SO4)2 x 12 H2O	0.02 g/L
H3BO3	0.01 g/L
Na2MoO4 x 2 H2O	0.01 g/L
NiCl2 x 6 H2O	0.03 g/L
Na2SeO3 x 5 H2O	0.30 mg/L
Vitamin solution:	
Biotin	2.00 mg/L
Folic acid	2.00 mg/L
Pyridoxine-HCl	10.00 mg/L
Thiamine-HCl x 2 H2O	5.00 mg/L
Riboflavin	5.00 mg/L
Nicotinic acid	5.00 mg/L
D-Ca-pantothenate	5.00 mg/L
Vitamin B12	0.10 mg/L
p-Aminobenzoic acid	5.00 mg/L
Lipoic acid	5.00 mg/L
Haemin solution	
Dissolve 50 mg haemin in 1 ml 1 N NaOH; make up to 100 ml with distilled water. Store refrigerated.	
Vitamin K1 solution	
Dissolve 0.1 ml of vitamin K1 in 20 ml 95% ethanol and filter sterilize. Store refrigerated in a brown bottle.	
Others	
acetic acid	33 mM
propionic acid	8 mM
butyric acid	4 mM

Medium41. GMM medium			
**Component**	**Amount/L**	**Concentration**	**Comments**
Tryptone Peptone	2 g	0.2%	
Yeast Extract	1 g	0.1%	
D-glucose	0.4 g	2.2 mM	
L-cysteine	0.5 g	3.2 mM	
Cellobiose	1 g	2.9 mM	
Maltose	1 g	2.8 mM	
Fructose	1 g	2.2 mM	
Meat Extract	5 g	0.5%	
KH2PO4	100 mL	100 mM	1M stock solution pH 7.2
$MgSO4\text{-}7H_20$	0.002 g	0.008 mM	
NaHCO3	0.4 g	4.8 mM	
NaCl2	0.08 g	1.37 mM	
CaCl2	1 mL	0.80%	0.8g/100mL stock
Vitamin K (menadione)	1 mL	5.8 mM	1 mg/mL stock solution
FeSO4	1 mL	1.44 mM	0.4 mg $FeSO_4$/mL stock solution
Histidine Hematin Solution	1 mL	0.1%	1.2 mg hematin/mL in 0.2M histidine
Tween 80	2 mL	0.05%	25% stock solution
ATCC Vitamin Mix	10 mL	1%	
ATCC Trace Mineral Mix	10 mL	1%	
Acetic acid	1.7 mL	30 mM	
Isovaleric acid	0.1 mL	1 mM	
Propionic acid	2 mL	8 mM	
Butyric acid	2 mL	4 mM	
Resazurin	4 mL	4 mM	0.25 mg/mL stock solution
Noble Agar	12 g	1.2%	

Medium42. GMM medium with mucin			
**Component**	**Amount/L**	**Concentration**	**Comments**
Tryptone Peptone	2 g	0.2%	
Yeast Extract	1 g	0.1%	
D-glucose	0.4 g	2.2 mM	
L-cysteine	0.5 g	3.2 mM	
Cellobiose	1 g	2.9 mM	
Maltose	1 g	2.8 mM	
Fructose	1 g	2.2 mM	
Meat Extract	5 g	0.5%	
KH2PO4	100 mL	100 mM	1M stock solution pH 7.2
$MgSO4\text{-}7H_20$	0.002 g	0.008 mM	
NaHCO3	0.4 g	4.8 mM	
NaCl2	0.08 g	1.37 mM	
CaCl2	1 mL	0.80%	0.8g/100mL stock
Vitamin K (menadione)	1 mL	5.8 mM	1 mg/mL stock solution
FeSO4	1 mL	1.44 mM	0.4 mg $FeSO_4$/mL stock solution
Histidine Hematin Solution	1 mL	0.1%	1.2 mg hematin/mL in 0.2M histidine
Tween 80	2 mL	0.05%	25% stock solution
ATCC Vitamin Mix	10 mL	1%	
ATCC Trace Mineral Mix	10 mL	1%	
Acetic acid	1.7 mL	30 mM	
Isovaleric acid	0.1 mL	1 mM	
Propionic acid	2 mL	8 mM	
Butyric acid	2 mL	4 mM	
Resazurin	4 mL	4 mM	0.25 mg/mL stock solution
Noble Agar	12 g	1.2%	
**Conponents that need to be added**			
**Component**	**Concentration**		
Mucin	4 g/L		

Medium43. GMM medium with DSS			
**Component**	**Amount/L**	**Concentration**	**Comments**
Tryptone Peptone	2 g	0.2%	
Yeast Extract	1 g	0.1%	
D-glucose	0.4 g	2.2 mM	
L-cysteine	0.5 g	3.2 mM	
Cellobiose	1 g	2.9 mM	
Maltose	1 g	2.8 mM	
Fructose	1 g	2.2 mM	
Meat Extract	5 g	0.5%	
KH2PO4	100 mL	100 mM	1M stock solution pH 7.2
MgSO4-7$H_2$0	0.002 g	0.008 mM	
NaHCO3	0.4 g	4.8 mM	
NaCl2	0.08 g	1.37 mM	
CaCl2	1 mL	0.80%	0.8g/100mL stock
Vitamin K (menadione)	1 mL	5.8 mM	1 mg/mL stock solution
FeSO4	1 mL	1.44 mM	0.4 mg $FeSO_4$/mL stock solution
Histidine Hematin Solution	1 mL	0.1%	1.2 mg hematin/mL in 0.2M histidine
Tween 80	2 mL	0.05%	25% stock solution
ATCC Vitamin Mix	10 mL	1%	
ATCC Trace Mineral Mix	10 mL	1%	
Acetic acid	1.7 mL	30 mM	
Isovaleric acid	0.1 mL	1 mM	
Propionic acid	2 mL	8 mM	
Butyric acid	2 mL	4 mM	
Resazurin	4 mL	4 mM	0.25 mg/mL stock solution
Noble Agar	12 g	1.2%	
**Conponents that need to be added**			
**Component**	**Amount/L**		
DSS	1 g		

Medium44. GMM medium with acetic acid			
**Component**	**Amount/L**	**Concentration**	**Comments**
Tryptone Peptone	2 g	0.2%	
Yeast Extract	1 g	0.1%	
D-glucose	0.4 g	2.2 mM	
L-cysteine	0.5 g	3.2 mM	
Cellobiose	1 g	2.9 mM	
Maltose	1 g	2.8 mM	
Fructose	1 g	2.2 mM	
Meat Extract	5 g	0.5%	
KH2PO4	100 mL	100 mM	1M stock solution pH 7.2
MgSO4-7$H_2$0	0.002 g	0.008 mM	
NaHCO3	0.4 g	4.8 mM	
NaCl2	0.08 g	1.37 mM	
CaCl2	1 mL	0.80%	0.8g/100mL stock
Vitamin K (menadione)	1 mL	5.8 mM	1 mg/mL stock solution
FeSO4	1 mL	1.44 mM	0.4 mg $FeSO_4$/mL stock solution
Histidine Hematin Solution	1 mL	0.1%	1.2 mg hematin/mL in 0.2M histidine
Tween 80	2 mL	0.05%	25% stock solution
ATCC Vitamin Mix	10 mL	1%	
ATCC Trace Mineral Mix	10 mL	1%	
Acetic acid	1.7 mL	30 mM	
Isovaleric acid	0.1 mL	1 mM	
Propionic acid	2 mL	8 mM	
Butyric acid	2 mL	4 mM	
Resazurin	4 mL	4 mM	0.25 mg/mL stock solution
Noble Agar	12 g	1.2%	
**Conponents that need to be added**			
**Component**	**Concentration**		
acetic acid	33 mM		

Medium45. GMM medium with acetic acid, DSS and mucin			
**Component**	**Amount/L**	**Concentration**	**Comments**
Tryptone Peptone	2 g	0.2%	
Yeast Extract	1 g	0.1%	
D-glucose	0.4 g	2.2 mM	
L-cysteine	0.5 g	3.2 mM	
Cellobiose	1 g	2.9 mM	
Maltose	1 g	2.8 mM	
Fructose	1 g	2.2 mM	
Meat Extract	5 g	0.5%	
KH2PO4	100 mL	100 mM	1M stock solution pH 7.2
MgSO4-7$H_2$0	0.002 g	0.008 mM	
NaHCO3	0.4 g	4.8 mM	
NaCl2	0.08 g	1.37 mM	
CaCl2	1 mL	0.80%	0.8g/100mL stock
Vitamin K (menadione)	1 mL	5.8 mM	1 mg/mL stock solution
FeSO4	1 mL	1.44 mM	0.4 mg $FeSO_4$/mL stock solution
Histidine Hematin Solution	1 mL	0.1%	1.2 mg hematin/mL in 0.2M histidine
Tween 80	2 mL	0.05%	25% stock solution
ATCC Vitamin Mix	10 mL	1%	
ATCC Trace Mineral Mix	10 mL	1%	
Acetic acid	1.7 mL	30 mM	
Isovaleric acid	0.1 mL	1 mM	
Propionic acid	2 mL	8 mM	
Butyric acid	2 mL	4 mM	
Resazurin	4 mL	4 mM	0.25 mg/mL stock solution
Noble Agar	12 g	1.2%	
**Conponents that need to be added**			
**Component**	**Concentration**		
acetic acid	33 mM		
DSS	1 g/L		
Mucin	4 g/L		

Medium46. GMM medium with sulfate			
**Component**	**Amount/L**	**Concentration**	**Comments**
Tryptone Peptone	2 g	0.2%	
Yeast Extract	1 g	0.1%	
D-glucose	0.4 g	2.2 mM	
L-cysteine	0.5 g	3.2 mM	
Cellobiose	1 g	2.9 mM	
Maltose	1 g	2.8 mM	
Fructose	1 g	2.2 mM	
Meat Extract	5 g	0.5%	
KH2PO4	100 mL	100 mM	1M stock solution pH 7.2
MgSO4-7$H_2$0	0.002 g	0.008 mM	
NaHCO3	0.4 g	4.8 mM	
NaCl2	0.08 g	1.37 mM	
CaCl2	1 mL	0.80%	0.8g/100mL stock
Vitamin K (menadione)	1 mL	5.8 mM	1 mg/mL stock solution
FeSO4	1 mL	1.44 mM	0.4 mg $FeSO_4$/mL stock solution
Histidine Hematin Solution	1 mL	0.1%	1.2 mg hematin/mL in 0.2M histidine
Tween 80	2 mL	0.05%	25% stock solution
ATCC Vitamin Mix	10 mL	1%	
ATCC Trace Mineral Mix	10 mL	1%	
Acetic acid	1.7 mL	30 mM	
Isovaleric acid	0.1 mL	1 mM	
Propionic acid	2 mL	8 mM	
Butyric acid	2 mL	4 mM	
Resazurin	4 mL	4 mM	0.25 mg/mL stock solution
Noble Agar	12 g	1.2%	
**Conponents that need to be added**			
**Component**	**Concentration**		
Na2SO4	0.1 g/L		
FeSO4	0.05 g/L		
(NH4)2SO4	0.09 g/L		
